# Heterotopic bone formation in the musculus latissimus dorsi of sheep using β-tricalcium phosphate scaffolds: evaluation of different seeding techniques

**DOI:** 10.1093/rb/rbx029

**Published:** 2017-11-27

**Authors:** Simon Spalthoff, Rüdiger Zimmerer, Jan Dittmann, Horst Kokemüller, Marco Tiede, Laura Flohr, Philippe Korn, Nils-Claudius Gellrich, Philipp Jehn

**Affiliations:** 1 Department of Oral and Maxillofacial Surgery, Hannover Medical School, Carl-Neuberg-Str. 1, Hannover 30625, Germany and; 2 Department of Conservative Dentistry, Periodontology and Preventive Dentistry, Hannover Medical School, Carl-Neuberg-Str. 1, Hannover 30625, Germany

**Keywords:** heterotopic bone formation, β-tricalcium phosphate, bone marrow stromal cells, bone marrow aspirate concentrate, prevascularization, ceramic degradation

## Abstract

Osseous reconstruction of large bone defects remains a challenge in oral and maxillofacial surgery. In addition to autogenous bone grafts, which despite potential donor-site mobility still represent the gold standard in reconstructive surgery, many studies have investigated less invasive alternatives such as *in vitro* cultivation techniques. This study compared different types of seeding techniques on pure β-tricalcium phosphate scaffolds in terms of bone formation and ceramic resorption *in vivo*. Cylindrical scaffolds loaded with autologous cancellous bone, venous blood, bone marrow aspirate concentrate or extracorporeal *in vitro* cultivated bone marrow stromal cells were cultured in sheep on a perforator vessel of the musculus latissimus dorsi over a 6-month period. Histological and histomorphometric analyses revealed that scaffolds loaded with cancellous bone were superior at promoting heterotopic bone formation and ceramic degradation, with autogenous bone and bone marrow aspirate concentrate inducing *in vivo* formation of vital bone tissue. These results confirm that autologous bone constitutes the preferred source of osteoinductive and osteogenic material that can reliably induce heterotopic bone formation *in vivo*.

## Introduction

Autogenous bone grafts remain the gold standard in oro-maxillo-facial surgery for reconstruction of large segmental bone defects [1, 2]. In cases where infection or radiation has compromised vascularization at the defect site, microsurgical bone transfer is typically performed to increase the probability of undisturbed osseointegration [3–7]. However, harvesting autogenous bone grafts can lead to donor-site morbidity, with the risk increasing according to the amount of harvested bone [8–10]. To overcome these limitations, different allogenic and xenogenic bone replacement materials have been developed for and applied to clinical use [11, 12]. However, the reconstruction of large bone defects by allo- and xenografts, which would be most beneficial for the patient, is associated with a high complication rate [13]. Therefore, alternative methods with lower donor-site morbidity and complication rates are needed.

The prefabrication of bioartificial autogenous bone grafts using tissue engineering techniques constitutes a promising strategy for generating vital bone without the aforementioned risks [14–17]. There are three key elements to bioartificial bone formation: an adequate 3D matrix with good osteoconductive, and if possible, osteoinductive properties; a large number of osteogenic cells and a sufficient supply of oxygen and nutrition. Our preliminary experiments showed that β-tricalcium phosphate (β-TCP) is an ideal matrix material for adequate seeding [18, 19]. However, larger and more complex scaffolds require a sufficient blood supply to provide nutrition to osteogenic cells [20–23]. We previously demonstrated that extrinsic or intrinsic *in vivo* prevascularization reliably induces the formation of vital heterotopic bone, and confirmed that autogenous bone marrow (BM) comprises a reliable source of osteogenic cells and growth factors that can be readily harvested [21, 24].

However, it is unknown whether morbidity can be further reduced by using different osteogenic cells, or whether the osteoinduction capacity of β-TCP is sufficiently high to generate bone without the need for supporting cells and growth factors. To address these issues, in this study, we utilized our established experimental model in which sheep are implanted with β-TCP scaffolds loaded with different seeding materials including cancellous bone, venous blood, BM aspirate concentrate (BMAC) or extracorporeal *in vitro*-cultured BM stromal cells (BMSCs). We then compared the effects of different β-TCP scaffold seeding techniques on bone formation and ceramic degradation, focusing on whether the use of an *in vitro* tissue engineering technique such as extracorporeal cultivation of BMSCs constitutes a reliable alternative to using autogenous cancellous bone.

## Materials and methods

Animal experiments were carried out as described in our previous report [24]. Sterile cylinders (length × diameter = 25 × 14 mm) with a preformed central tunnel 7 mm in diameter consisting of pure β-TCP were used as carriers (chronOS; DePuy Synthes, USA). The protocol for animal experiments received ethical approval in accordance with German federal animal welfare legislation. Specifically, ethical approval for the experimental study in sheep was provided by the Department for Veterinary Affairs, Oldenburg, Germany (AZ: 08/1621). A total of 24 cylinders were randomly implanted into 18 healthy adult female German blackheaded sheep.

**Figure 1:**
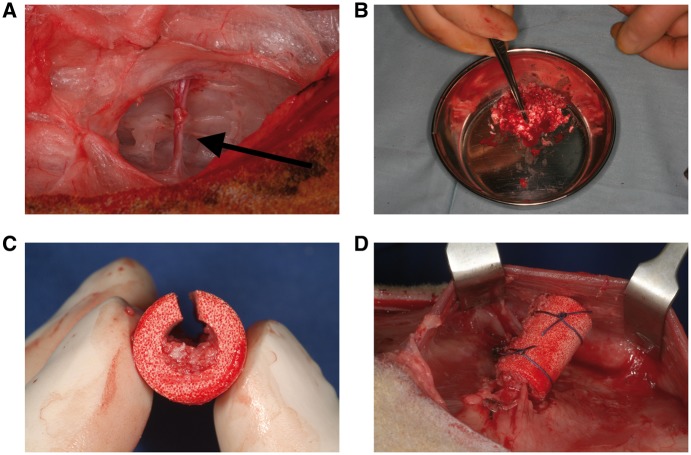
Surgical procedure employed in the study. (**a**) Exposed bottom side of the musculus latissimus dorsi showing the arterio-venous vessel bundle (arrow). (**b**) Morselized bone from the iliac crest. (**c**) Sliced cylinder from Group A partly filled with bone. (**d**) Cylinder implanted on the vessel bundle and fixed with sutures

In Group A (*n* = 6), three bone biopsies (5 mm in diameter) were harvested from the iliac crest and morselized using an electric bone mill. The morselized bone was mixed with amorphous BMAC obtained from deep donor sites and loaded into the central cavities of predrilled blood-soaked cylinders, which were then implanted underneath the musculus latissimus dorsi on an arterio-venous vessel bundle serving as a central perfusion line, such that the whole animal acted as a natural bioreactor (Fig. 1a–d).

**Figure 2:**
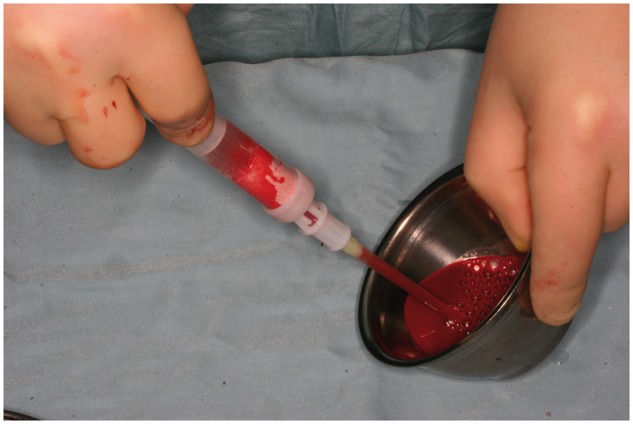
Cylinder from Group B soaked with venous blood using the chronOS kit

**Figure 3:**
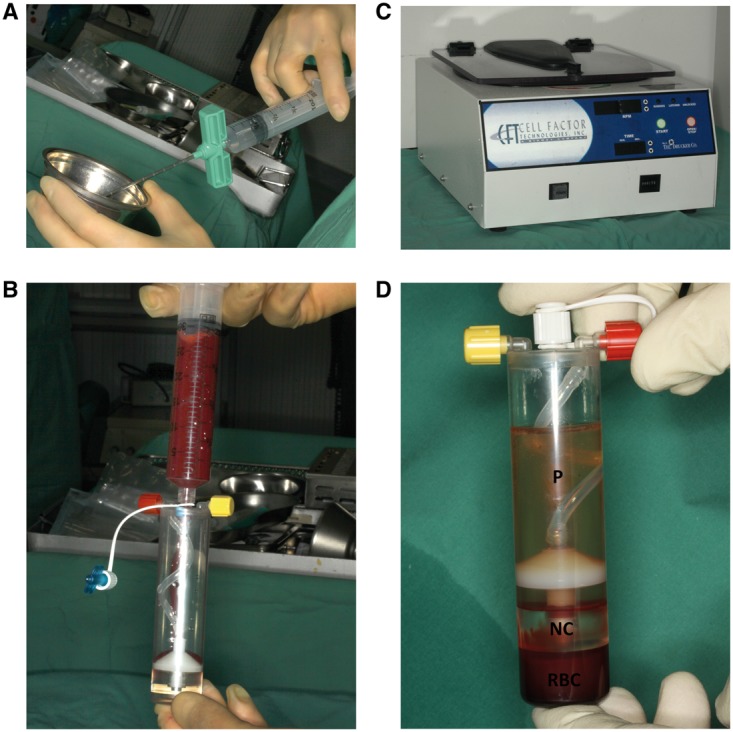
Preparation of BMAC. (**a**) Rinsing the aspiration needle with anticoagulant solution. (**b**) Separation chamber filled with BMAC. (**c**) Centrifuge used in the experiment. (**d**) Separated BMAC (NC, nucleated cell; P, plasma; RBC, red blood cell)

In Group B (*n* = 6), cylinders were soaked with venous blood collected from the vena jugularis using the chronOS kit and then implanted as described (Fig. 2). In Group C (*n* = 6), BMAC was obtained from the left iliac crest using the MarrowStim BM aspiration and concentration system (Biomet Biologics, USA). The aspiration needle was gently inserted into the BM and flushed with anticoagulants (Liquemin; Hoffmann-La Roche AG, Germany), followed by aspiration of BM into an attached syringe. The BM was transferred to a chamber that was centrifuged at 3200 rpm for 15 min (Biomet Biologics), which separated the aspirate into three clearly visible layers; from top to bottom, these comprised the acellular plasma, nucleated cells and red blood cells. The plasma was removed and the cellular fractions were resuspended (Fig. 3a–d). The concentrated BMAC was loaded onto prepared cylinders.

**Figure 4:**
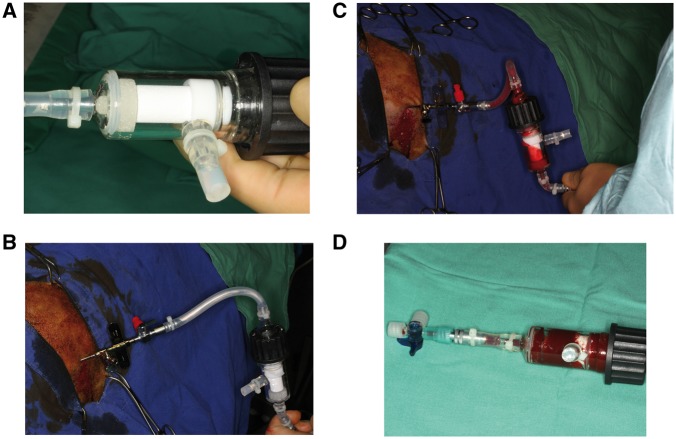
Seeding of BMSCs (**a**) cylinder from Group D inside the bioreactor. (**b**) Insertion of the Yamcidi aspiration needle into the iliac crest. (**c**) Aspiration of BM through the bioreactor. (**d**) Bioreactor containing the soaked cylinder

**Figure 5:**
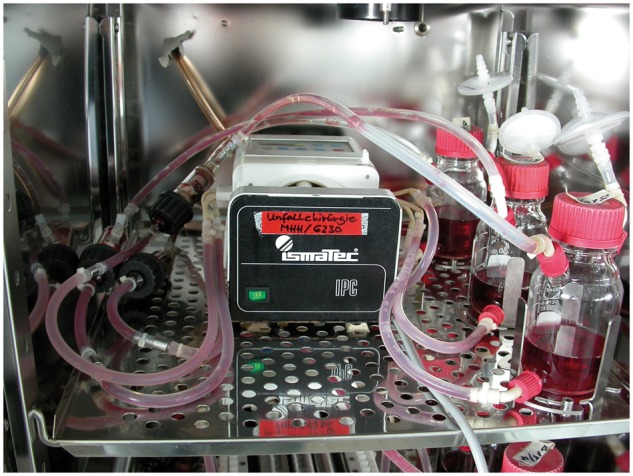
Bioreactor with the continuous flow perfusion system inside the incubator

In Group D (*n* = 6), BMSCs from the left iliac crest were aspirated through a cylinder within a custom-made bioreactor chamber. Briefly, a stab incision was made at the most prominent aspect of the iliac crest and a Yamcidi aspiration needle was inserted into the center of the marrow cavity to a depth of ∼5 cm. The marrow was aspirated directly through a silicone tube into the porous scaffold, a technique that has been previously used for bone tissue engineering [25]. The culture chamber contained the porous β-TCP scaffold along with an inflow and two outflow valves. For cell seeding, one outflow valve was closed and another was opened to allow the aspirate to disperse through the scaffold pores. The bioreactor was filled with saline containing heparin; the same solution was used to irrigate the aspiration syringe before use (Fig. 4a–d). After cell loading, the bioreactor was filled with an angiogenic medium as described later and incubated under continuous perfusion for 3 weeks (Fig. 5).

During incubation, the bioreactor was rotated at 5° min^−1^ for 24 h. The scaffold was perfused with 500 ml of angiogenic medium composed of Dulbecco’s modified Eagle’s medium/Ham’s F-12 (Biochrom, Germany), 10% fetal calf serum (Gibco, Germany), 100 U penicillin, 0.2 mg/ml streptomycin (Biochrom), 0.5 µg/ml amphotericin B (Biochrom), 5 µg/ml ascorbic acid (Sigma-Aldrich Chemie GmbH, Germany), 0.02 nM/ml dexamethasone (Merck, Germany) and 30 ng/ml of the Toll-like receptor-2/6 agonist macrophage-activating lipopeptide (MALP)-2 (Axxora GmbH, Germany) to induce angiogenesis. The bioreactor was incubated at 37 °C and culture was performed with a medium flow of 2 ml min^−1^ at 5% CO_2_. Every third day, 50 ml of culture medium was replaced with fresh medium. After the culture period ended, the bioreactor was disconnected and transferred to the operating room at 5 °C, and the cylinders were then implanted in the described manner.

At 6 months after the operation, animals were euthanized after deep sedation (1 mg/kg midazolam by intramuscular injection and 5 mg/kg propofol with 80 mg/kg pentobarbital by intravenous injection). Cylinders were removed from the latissimus dorsi muscle and fixed in 3.5% neutral buffered formalin, embedded in methylmethacrylate and sectioned perpendicular to the axis of the cylinder using a modified inner-hole diamond saw. Non-decalcified sections with a thickness of 30 mm were surface-stained with Alizarin-methylene blue for standard light microscopy and histomorphometric analyses. Digital images of each section were acquired using a Zeiss Axio Imager microscope fitted with an AxioCam MRc digital camera and AxioVision v.4.5 software (Carl Zeiss, Germany). The AxioVision module MosaiX was used to generate images of whole cylinder cross sections. Total bone and residual ceramic areas were quantified using analy-SIS v.3.0 image analysis software (Olympus Soft Imaging Solutions, Germany). A total of six sections distributed evenly throughout each cylinder were used for histomorphometric evaluations.

## Results

All animals survived surgery without any complications and were capable of ingestion and rumination immediately afterward. The site of implantation was examined daily and showed no signs of infection or rejection until animals were sacrificed. There was no postoperative swelling of the thoracic wall and no indication of compromised mobility.

**Figure 6:**
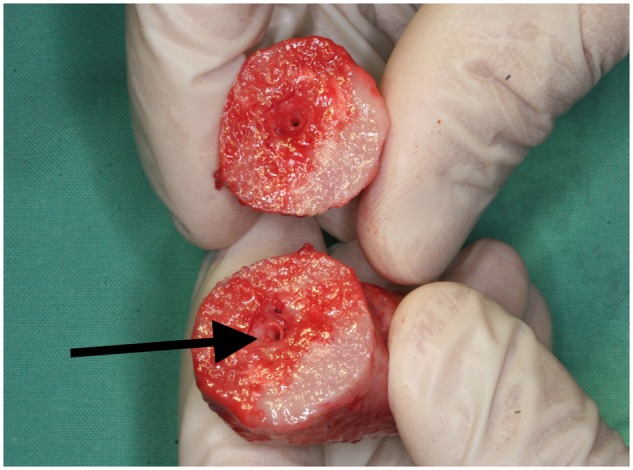
Sliced cylinder from Group A after explantation showing the intact blood vessel bundle (arrow) and vital bone

**Figure 7:**
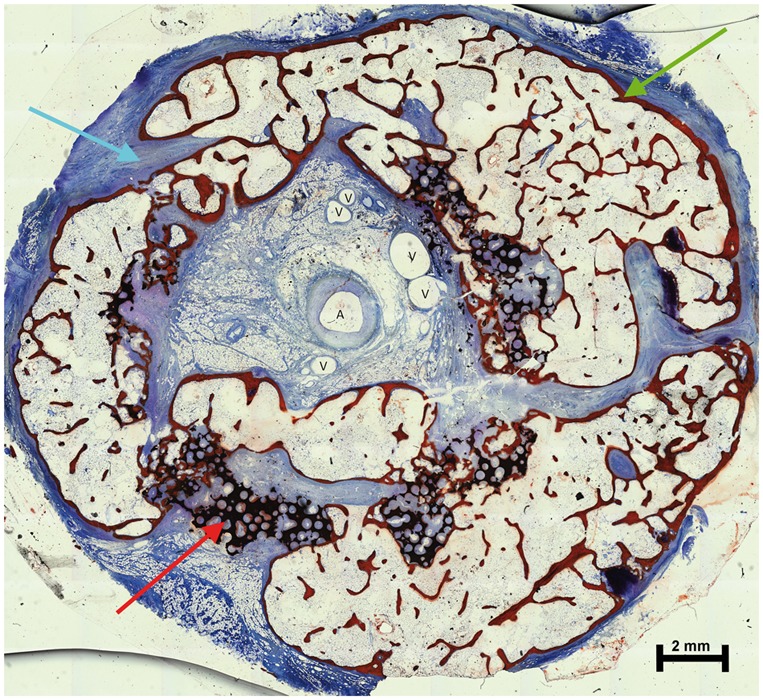
Cross section of a cylinder from Group A, surface-stained with Alizarin-methylene blue (red arrow, residual ceramic material; blue arrow, fibrous soft tissue; green arrow, bone; A, artery and V, vein)

**Figure 8:**
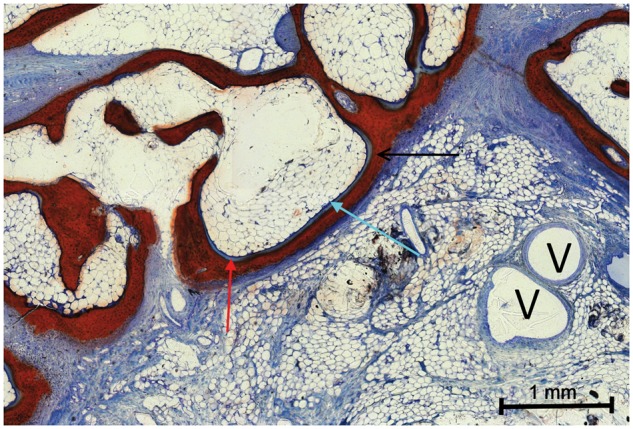
Magnification of Fig. 7 (red arrow, osteoid; blue arrow, osteoblast; black arrow, osteocytes with mature bone and V, vein)

Explanted constructs in Group A had a hard consistency with no flexibility. In all cylinders, the central vessel bundle was easily detectable after separation of the two halves (Fig. 6). There was bleeding throughout the surface, providing evidence for tissue regeneration. Bone formation was observed inside the cylinders by microscopy. A substantial portion of the β-TCP scaffolds was degraded and replaced with osseous tissue. Osteogenic activity was evidenced by the presence of osteoblasts and osteoids. Bony infiltration of β-TCP with conversion to bone was observed throughout the implant. Well-vascularized soft fibrous tissue had invaded the remaining parts of the matrix material (Figs 7 and 8).

**Figure 9:**
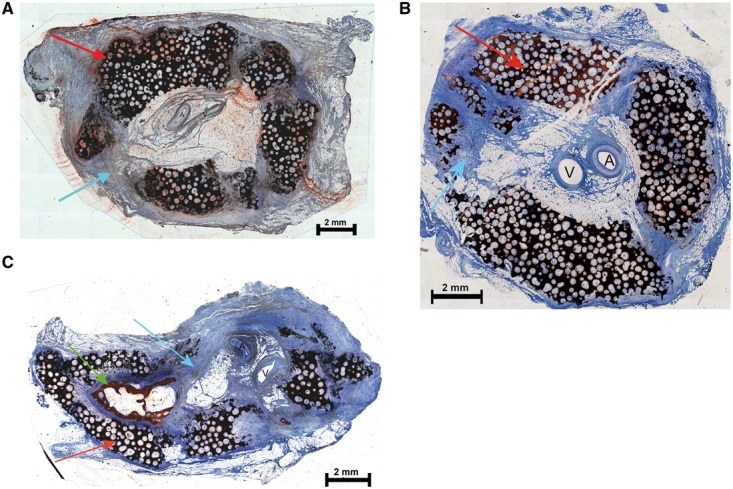
Histological analysis of cylindrical scaffolds. Cross section of a representative cylinder from Group B (**a**), Group C (**b**) and Group D (**c**). All sections were surface-stained with Alizarin-methylene blue (red arrow, residual ceramic material; blue arrow, fibrous soft tissue; green arrow, bone; A, artery and V, vein)

Only five cylinders were recovered from Group D. Examination by palpation revealed that the cylinders from Groups B–D were not rigid but instead exhibited 3D flexibility. After separation, the implanted vascular bundle was detectable in the soft fibrous tissue. A significant amount of residual ceramic material was macroscopically observed, suggesting that the cylinders had been crushed rather than degraded. Only a small amount of bone was observed in Group C by microscopy, whereas Groups B and D showed no signs of newly formed bone, consistent with the lack of stability of the implant (Fig. 9).

**Figure 10:**
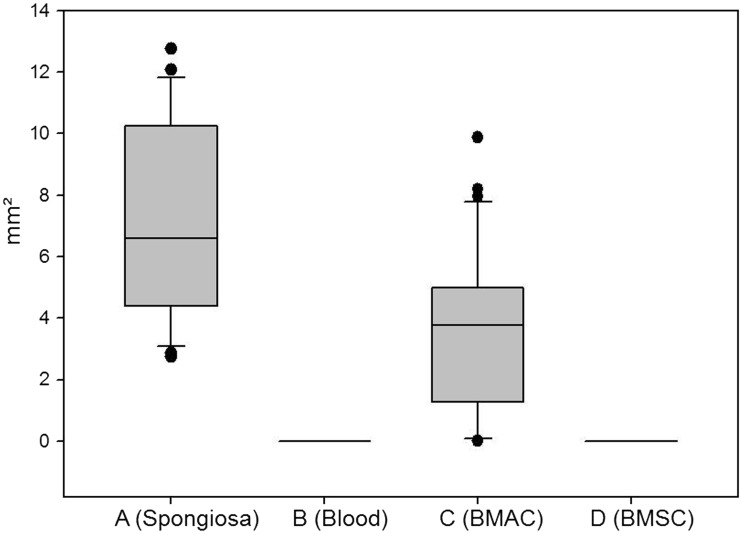
Bone area (mm^2^) in Groups A–D. For A–C, *n* = 6; for D, *n* = 5

**Figure 11:**
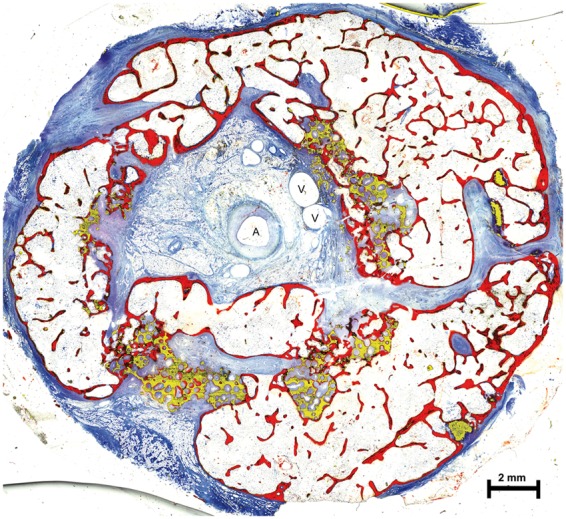
Exemplary cross section of the histomorphometric analysis of a cylinder from Group A (bone colored in red, residual ceramic material colored in yellow; A, artery and V, vein)

**Figure 12:**
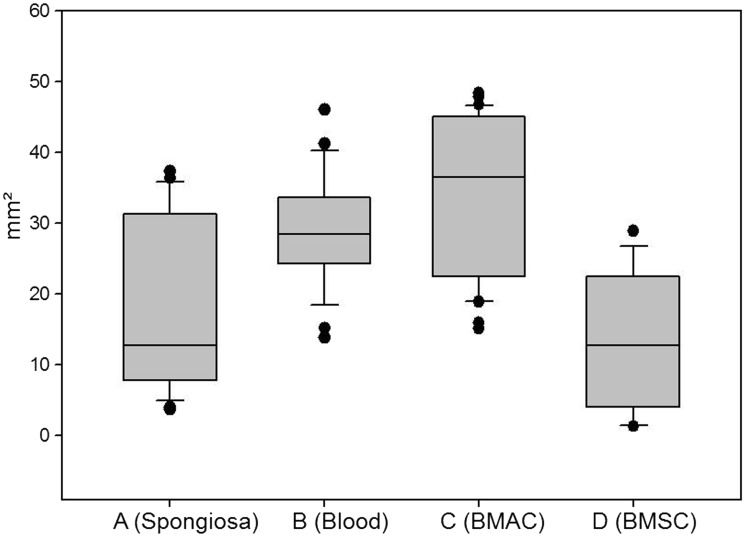
Area of residual ceramic (mm^2^) in Groups A–D. For A–C, *n* = 6; for D, *n* = 5

More bone formation was observed in Group A than in the other groups (Figs 10 and 11; Table 1). It was difficult to distinguish the effects of degradation and crushing; however, the cylinders from Group A appeared more degraded than those from the other groups (Fig. 12 and Table 2).

**Table 1:** Area of bone^a^

Group	Mean	SD	*P*
	mm²	mm²	
A (Spongiosa)	7.03	3.22	
B (Blood)	0	0	<0.001
C (BMAC)	3.57	2.50	<0.001
D (BMSC)	0	0	<0.001

^a^*T*-test of Groups B–D versus Group A. *P* < 0.001 indicates significantly more bone formation in Group A compared with the indicated group.

**Table 2:** Area of residual ceramic^a^

Group	Median	25%	75%	*P*
	mm²	mm²	mm²	
A (Spongiosa)	12.77	7.83	30.21	
B (Blood)	28.47	24.51	33.31	0.003
C (BMAC)	36.51	22.9	45.06	<0.001
D (BMSC)	12.77	4.13	21.72	0.16

^a^*P*, Rank sum test of Groups B–D versus Group A.

## Discussion

BMAC is widely used in modern regenerative medicine, such as for spinal fusion in elderly patients in combination with allogeneic bone chips and demineralized bone matrix, which achieved a very good solid fusion rate of 83.9% [26, 27]. BMAC has also been used in combination with β-TCP; e.g. tibia defects in rabbits were reconstructed with β-TCP loaded with platelet-rich plasma (PRP) or BMAC, with both methods showing bony consolidation of the defect, although this was greater in the PRP group [28, 29]. A combination of BMAC, PRP and calcium phosphate granules was used to reconstruct critical-size metaphyseal defects in the proximal tibia of miniature pigs and was found to be a reasonable alternative to autologous bone grafting [30]. In another study, bone defects were treated with a combination of BMAC and hydroxyapatite or collagen, which promoted full healing after 6 months [31]. Moreover, patients with lower jaw continuous defects after benign tumor resection in whom the missing bone was replaced by a combination of allogeneic bone, bone morphogenetic protein and BMAC showed bone regeneration of a sufficiently high quality to accommodate dental implants after 12 months [32].

In this study, we found that BMAC comprises an effective osteogenic and osteoinductive material that promotes heterotopic bone formation in combination with β-TCP. However, autologous cancellous bone from the iliac crest was still superior, as significantly more new bone was formed that exhibited bone-like behavior than had been observed with BMAC [33]. In particular, cancellous bone or BM constitutes a well-known donor of osteoinductive cells as BMSCs or mesenchymal stem cells exhibit a considerable ability to regenerate bone tissue [34, 35]. Cancellous bone or marrow also contains other osteoprogenitor cells and valuable bone matrices that induce ectopic bone formation [36]. Cancellous or autologous bone is therefore widely used in bone tissue engineering procedures to vitalize different matrix materials. BMSCs have also been used to vitalize scaffolds for bone tissue engineering and induced the formation of new bone when used in conjunction with various matrix materials and growth factors [37–44]. For example, a calcium phosphate matrix with BMSCs induced subcutaneous bone formation in mice, with similar heterotopic bone formation being demonstrated in a subcutaneous pocket in sheep using hydroxyapatite as the matrix material [45, 46]. Furthermore, continuous flow perfusion has been reported to have positive effects on osteoblasts, such as stimulation of osteoblast maturation and deposition of the mineralized matrix [47].

MALP-2 promotes early implant vascularization, which plays a critical role in bone growth and repair [44, 48–50]. In this study, we attempted to induce early vascularization *in vivo* to enhance the survival of BMSCs loaded onto the β-TCP scaffold; however, seeded BMSCs did not stimulate new bone formation. This may be because the number of seeded cells was too low or because our measurement method was not sufficiently sensitive to detect small numbers of osteoblasts and osteocytes present within or on the surface of the β-TCP cylinders. Ultimately, the constructs were not stable after 6 months and were therefore deemed ineffective. Calcium phosphate-based ceramics are known for their osteoinductive properties, although the type of ceramic, its micro and macro structures, and the animal model that is used influence the extent of heterotopic bone formation [51–55]. In this study, we used chronOS as a matrix material. This consists of highly purified β-TCP with interconnected macro and micro pores, which would presumably induce heterotopic bone formation without the need for additional osteoinductive materials. Conversely, in our experimental model, there was no heterotopic bone formation induced by blood-soaked cylinders. This phenomenon has mostly been described in dogs, rabbits and mice; in contrast, bone formation has not been successfully induced in rats. It is therefore possible that sheep are also not a suitable model for heterotopic bone formation in muscle tissue [56].

In conclusion, cancellous bone was a reliable inducer of osteoinduction in our experimental model; however, BMAC was less effective, whereas β-TCP alone or in combination with BMSCs showed no capacity to induce heterotopic bone formation in the musculus latissimus dorsi of sheep. Future studies will investigate whether the addition of growth factors such as bone morphogenetic proteins, potentially administered via a sustained release system, might improve heterotopic bone formation in our experimental model [39, 57]. 
